# Home Biofilm Management in Orthodontic Aligners: A Systematic Review

**DOI:** 10.3390/dj12100335

**Published:** 2024-10-21

**Authors:** Alessia Pardo, Annarita Signoriello, Alessandro Zangani, Elena Messina, Selene Gheza, Paolo Faccioni, Massimo Albanese, Giorgio Lombardo

**Affiliations:** Section of Oral and Maxillofacial Surgery, Department of Surgical Sciences, Dentistry, Gynecology and Pediatrics, University of Verona, 37124 Verona, Italy; alessia.pardo@univr.it (A.P.); elena.messina@univr.it (E.M.); selene.gheza@studenti.univr.it (S.G.); paolo.faccioni@univr.it (P.F.); massimo.albanese@univr.it (M.A.); giorgio.lombardo@univr.it (G.L.)

**Keywords:** bacterial loads, cleaning, clear aligners, removable appliances, oral hygiene

## Abstract

**Background.** Transparent aligners are recently introduced orthodontic devices considered promising for the improvement of oral health conditions, in terms of faster treatment times and enhanced comfort, especially if compared with traditional fixed orthodontic therapy. This systematic review aimed to evaluate at-home protocols for proper oral hygiene and aligners cleaning during orthodontic treatment. **Methods.** A search was conducted using the following four databases: PubMed, Cochrane Library, Web of Science, and Scopus. The systematic review (registered as CRD 42024562215) followed the Preferred Reporting Items for Systematic Reviews and Meta-Analyses (PRISMA) 2020 guidelines and included prospective studies, randomized controlled trials (RCTs), controlled clinical trials, and in vivo and ex vivo studies; they had to assess treatment with invisible orthodontics compared to treatment with fixed orthodontics, home oral hygiene, or aligner disinfection protocols. The evidence in the studies was evaluated for risk of bias using the RoB-2 (for RCTs and randomized crossover studies) and ROBINS-I tools (for observational studies). **Results.** Eleven studies were included in this systematic review: four RCTs, four crossover studies, and three cross-sectional observational studies. Seven studies considered patients undergoing orthodontic treatment, whereas four examined orthodontic aligners. The cleaning protocols of the aligners were evaluated based on the analysis of residual biofilm on the thermoplastic surfaces. Studies included were characterized by a low level of certainty, thus further evidence is needed. **Conclusions.** The most effective protocols entailed a combination of mechanical and chemical agents, suggesting that it is fundamental for patients undergoing aligner treatment to focus on individually tailored home oral hygiene protocols.

## 1. Introduction

Orthodontic treatment with aligners represents an increasingly used type of intervention. Its recent popularity includes benefits such as faster treatment times and enhanced comfort. The widespread use of aligners is related to the current tendency of society toward aesthetics and harmony canons, to which contemporary dental treatments are simultaneously adapting. This is in line, at the same time, with the emerging demand for orthodontic treatments combining functional and clinical motivations with aesthetic requests [1].

Recent surveys [2] confirm this concept, as 70% of participants declared to be exactly driven by aesthetic reasons to initiate orthodontic treatment [2], evidencing that the popularity of orthodontic treatment using aligners overcomes traditional fixed orthodontics, in terms of better aesthetics and easier at-home oral hygiene procedures after their removal. Nevertheless, issues regarding the condition of soft tissues during aligner treatment are not yet well known. In this regard, proper specific oral hygiene maneuvers are essential, because of the material’s micro-roughness and the almost overall daily contact with the dental elements: extensive plaque accumulation occurs if they are not properly cleaned and sterilized [3]. In addition, adequate and precise cleansing of the oral cavity is necessary, as natural mechanisms of self-deterrence and protection, given by the movement of the tongue and lips over the teeth, as well as by the buffering effect of saliva, are reduced or lacking, because of the obstacle represented by aligners [4].

Orthodontic appliances are defined as foreign surfaces that create an ideal condition for biofilm formation [5], usually presenting specific features depending on the surrounding environment. Biofilm growing conditions, in terms of colonizing surface morphology and roughness, and oxygen or substrate availability influence all interactions regarding bacterial species and biofilm matrix [6]. The composition of long-term supra-gingival plaque related to orthodontic treatment mostly comprehends facultative anaerobic bacteria, with a predominant cariogenic role (e.g., *Streptococcus mutans*, *salivarius*, and *sobrinus* as Gram-positive), and a less prevalent periodontal role (e.g., *Aggregatibacter actinomycetemcomitans* as Gram-negative) [3].

Even if it was suggested [7] that clear aligners may be more beneficial for periodontal health compared to fixed appliances in limiting plaque accumulation and excluding bacterial affinity to metallic surfaces, data regarding the characterization of oral microflora of these devices are still limited. In patients treated with fixed appliances, anaerobic bacteria were specifically found on the enamel surfaces [8], with higher levels of *Flavobacteriaceae* (e.g., *Capnocytophaga sputigena* as Gram-negative, usually involved in periodontal inflammation and infection), *Prevotellaceae* (e.g., *Prevotella intermedia* as anaerobic species with a role in proteins and carbohydrates breakdown, with prevalent association with periodontal disease), and *Sacchariomonadaceae* (reported in cases of oral mucosal infection). 

On the other hand, elevated levels of *Burkholderiaceae* (e.g., *Burkholderia cepacia*, a Gram-negative bacterium not commonly associated with the oral cavity, but typically found in human pulmonary infections of cystic fibrosis) were found in patients with aligners [8]. As alterations of the oral environment can lead to changes in the composition or metabolic activity of the oral microbiome [9], bacteria present in patients treated with aligners seem to process amino acids differently compared to the normal oral microbiome, due to changes in pH, oxygen levels, or nutrient availability caused by the aligners. It has been shown that the salivary and teeth surface microbiota of patients with aligners undergo changes 12 h post-application of the device [10].

Clear aligners are removable and fit over the teeth thanks to their thermoplastic material, which itself may influence the oral environment and affect bacterial growth differently than traditional braces [11]. Moreover, invisible braces fully envelop the tooth surface, whereas tooth surfaces during fixed orthodontic treatment are more exposed to the oral environment [8]. The alteration in the microbiome composition, particularly the increase in families associated with periodontal pathogens, underscores the importance of enhanced and individually tailored oral hygiene practices, both for fixed and removable devices [12]. 

In this proposal, the existing knowledge refers to current systematic reviews [4], which focus on key aspects of procedures only for aligner cleansing, such as brushing with toothpaste or vibration, chemical methods/pharmaceutical products (chlorhexidine antibacterial substance, anionic or cationic detergents or effervescent tablets), or combinations of both. Nevertheless, further considerations concerning protocols for overall home oral hygiene during the entire treatment are needed, especially considering that procedures effective for fixed devices are not as valid as for removable ones, for all aspects abovementioned described in terms of biofilm and plaque characterization.

In light of these considerations, this systematic literature review aimed to investigate the most effective home oral hygiene practices for preserving dental and periodontal tissues during aligner treatment.

## 2. Materials and Methods

This systematic review was conducted in accordance with the PRISMA guidelines and registered in the International Prospective Register of Systematic Reviews (PROSPERO) CRD 42024562215.

The PICOS criteria were set as follows:

- Patients: healthy patients wearing invisible orthodontics;
- Interventions: home hygiene protocols;
- Comparators: negative control or placebo;
- Outcomes: presence of bacterial biofilm;
- Study design: prospective studies, randomized controlled trials, and controlled clinical trials, published in English in the past two decades, evaluating the efficacy of home hygiene protocols on the patient or template, were included.

### 2.1. Eligibility Criteria and Focused Question

Studies eligible for inclusion in this review were prospective studies, randomized controlled clinical trials (RCTs), controlled clinical studies, and in vivo and ex vivo studies; they had to assess treatment with invisible orthodontics, treatment with invisible orthodontics compared to treatment with fixed orthodontics, home oral hygiene maintenance protocols, or home disinfection protocols for aligners.

Excluded were systematic reviews, case reports and case series, studies on animals, in vitro studies, and studies focusing on interventions with orthodontic surgery or treatment exclusively with fixed orthodontics. Studies that did not assess home oral hygiene protocols were also excluded.

The focus questions were: “How to maintain proper oral hygiene at home during treatment with invisible orthodontics? How to keep aligners cleansed during treatment with invisible orthodontics?”

### 2.2. Search Strategy

An electronic search was implemented to retrieve all relevant studies. The search was performed using the PubMed, Cochrane Library, Scopus, and Web of Science databases. A search string was entered in each of the above four databases; for PubMed, Cochrane Library, and Web of Science, the same string was used, while for Scopus a special string was created.

Relevant keywords and Boolean operators (AND, OR, NOT) were used to implement the following search string: (clear aligners OR Invisalign OR removable appliances OR clear orthodontic aligner OR acrylic baseplates) AND (oral hygiene OR bacterial loads OR contaminations) OR cleaning (methods OR tablets) OR oral colonisation OR biofilm adherence) for PubMed, Cochrane Library, and Web of Science; for Scopus, the search string was clear AND aligners OR removable OR Invisalign AND appliances AND oral AND hygiene OR cleaning AND methods.

### 2.3. Screening and Selection

Two independent reviewers (AP, SG) reviewed the records obtained from the search. The selection process included the removal of duplicates as the first step, followed by the elimination of articles that were not accessible, published in languages other than English, or before 2003. The titles and abstracts of the remaining records were analyzed, and those not relevant to the inclusion criteria were excluded. Finally, the full text of the studies considered most relevant, as a result of the previous steps, was read to assess their inclusion in the review. In case of disagreement between the two reviewers, a third party (AS) intervened in the decision-making process. Data collection from the included reports was performed by the first two reviewers (AP, SG), who independently worked to identify parameters referring to general article information (title, author, year of publication, journal, volume, and pages), study design, the population included, type of intervention, control used, and outcomes. Data collection, extraction, and management were performed with Review Manager (RevMan) Version 5.4 for Windows. Copenhagen: The Nordic Cochrane Center, Cochrane Collaboration, 2003.

### 2.4. Risk of Bias Assessment

The methodological quality of the included studies was analyzed using the risk of bias assessment performed by two independent reviewers (AP, SG). In case of disagreement, a third one (AS) intervened in the decision-making process. An adequate tool for proper bias risk assessment was selected based on the type of included studies as follows. For randomized controlled trials (RCTs) and randomized crossover studies, the appropriate Cochrane Rob-2 tool was used [13], while the Cochrane ROBINS-I tool was used for cross-sectional observational studies [14].

## 3. Results

**Figure 1:**
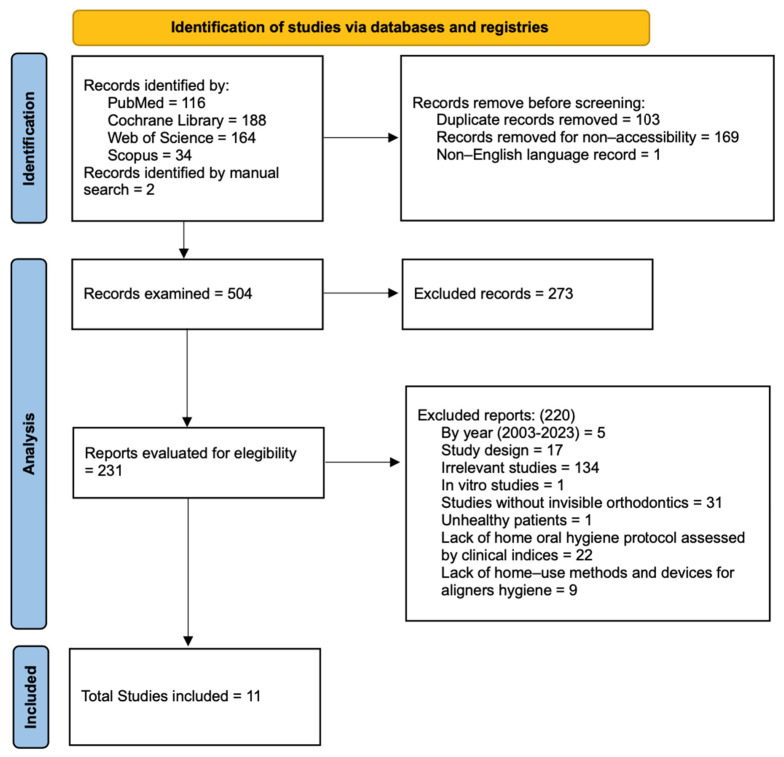
Study selection. Flowchart based on the PRISMA 2020 model [24].

The complete selection process, starting from the results obtained with the search string in each database to the records selected for inclusion, is shown as a flowchart in Figure 1.

First, 502 articles were obtained from the digital search (PubMed 116; Cochrane Library 188; Web of Science 164; Scopus 34). Two reports were added using a manual citation search. A total of 273 records were removed prior to screening: 103 duplicates, 169 inaccessible records, and 1 article published in a language other than English. Relevance analysis by title and abstract involved 231 articles, and 220 were eliminated. Of these, 5 were excluded by year of publication, 18 by study design not relevant to the inclusion criteria, 134 by irrelevance, 31 by lack of treatment with invisible orthodontics, 1 by inclusion of subjects with pathology, and 31 by lack of home hygiene protocols (22 did not report home oral hygiene protocols on the patient; 9 did not report aligner disinfection protocols).

Therefore, nine reports were selected for inclusion in the review after reading the full text of the article. Of the nine included studies, four were identified from the PubMed platform [15,16,17,18], one from the Cochrane Library [19], and four from the Web of Science database [20,21,22,23], whereas no reports identified by Scopus were included in the review. These nine identified by means of electronic databases were added to the two studies included in the manual literature search. Eleven studies were considered finally eligible for review.

### 3.1. Characteristics of Included Studies

The main characteristics of the studies included in this review are summarized in Table 1. Of the 11 articles included, 4 were randomized controlled trials (RCTs), 4 were crossover studies, and 3 were cross-sectional observational studies. The publication dates ranged from 2013 to 2022 for all records considered. Seven studies considered patients with invisible orthodontics or patients divided between invisible and fixed orthodontics as the population. Four studies examined aligners, with 3–12 pairs of aligners per patient. Among the included publications, seven investigated the effectiveness of home oral hygiene protocols on patients with invisible orthodontics and compared the effectiveness of protocols on patients with invisible orthodontics to patients with fixed orthodontics, with or without a control group. Four studies evaluated the usefulness of different cleansing and disinfection techniques for templates. For the seven clinical studies, the results were examined using periodontal indices and/or microbiological analysis. Plaque index (PI), gingival index (GI), bleeding on probing (BoP), and probing pocket depth (PPD) were the most frequently found outcomes. The outcomes of the four extraclinical studies were represented by microbiological analysis. Scanning electron microscopy (SEM), photodensitometry, and ATP analysis were performed using a bioluminometer.

**Table 1:** Description of the included studies.

Author	Year	Journal	Study Design	Intervention Population	Intervention	Outcome
Azaripour et al. [22]	2015	*BMC Oral Health*	Cross-sectional study	100 participants; 50 with invisible orthodontics, 50 with fixed orthodontics.	Patient home hygiene protocol	Gingival index; sulcus bleeding index; approximal plaque index
Caccianiga et al. [22]	2022	*Healthcare*	Cross-sectional observational study	50 participants; 25 with invisible orthodontics, 25 with fixed orthodontics.	Patient home hygiene protocol	Qualitative microbiological analysis of oral flora: pathogenic or non-pathogenic. Plaque assessment by SEM
Chhibber et al. [19]	2018	*American Journal of Orthodontics and Dentofacial Orthopedics*	Randomized control trial (RCT)	61 participants; 24 with invisible orthodontics, 37 with fixed orthodontics (17 with self-ligating attachments and 20 with elastomeric attachments).	Patient home hygiene protocol	Plaque index; gingival index; papillary bleeding index
Levrini et al. [25]	2013	*Cumhuriyet Dent J*	Randomized control trial (RCT)	30 participants; 10 with invisible orthodontics, 10 with fixed orthodontics, 10 without orthodontics (control group).	Patient home hygiene protocol	Plaque index; probing pocket depth; bleeding on probing; microbiological analysis through real-time PCR evaluation
Levrini et al. [26]	2015	*European Journal of Dentistry*	Randomized control trial (RCT)	77 participants; 32 with invisible orthodontics, 35 with fixed orthodontics, 10 without orthodontics (control group).	Patient home hygiene protocol	Plaque index; probing pocket depth; bleeding on probing; microbiological analysis through real-time PCR evaluation
Levrini et al. [18]	2015	*Clinical*, *Cosmetic and Investigational Dentistry*	Crossover study	36 aligners (3 aligners for 12 patients).	Orthodontic devices home hygiene protocol	Microbiological analysis: evaluation of the amount of plaque by SEM
Levrini et al. [16]	2016	*International Journal of Dentistry*	Crossover study	36 aligners (3 aligners for 12 patients).	Orthodontic devices home hygiene protocol	Microbiological analysis: evaluation of bacterial concentration by analyzing the amount of ATP using a bioluminometer. Bacterial concentration is expressed in RLUs (relative light units)
Lombardo et al. [17]	2017	*Progress in Orthodontics*	Crossover study	45 aligners (9 aligners for 5 patients).	Orthodontic devices home hygiene protocol	Microbiological analysis: observation of the presence of biofilms by scanning electron microscopy (SEM). The measurement is carried out with a Grey scale
Sfondrini et al. [21]	2021	*Applied sciences*	Randomized control trial (RCT)	40 participants; 20 with invisible orthodontics, 20 without orthodontics (control group).	Patient home hygiene protocol	Plaque index; bleeding on probing; probing pocket depth; quantitative and qualitative microbiological analysis of the flora through real-time PCR analysis
Shpack et al. [15]	2014	*Angle Orthodontist*	Crossover study	132 aligners (12 aligners for 11 patients).	Orthodontic devices home hygiene protocol	Microbiological analysis: evaluation of biofilm adhesion measured by photodensitometer
Zhao et al. [20]	2020	*Oral Diseases*	Cross-sectional study	25 with invisible orthodontics.	Patient home hygiene protocol	Plaque index; probing pocket depth; bleeding on probing

### 3.2. Main Outcome of the Study

The outcomes of the included studies were divided based on the type of protocol presented: the results of publications concerning aligner cleansing and disinfection are visible in Table 2, and those concerning patients’ at-home oral hygiene protocols are in Table 3.

All four articles in the first group evaluated the quality and bacterial adhesion to the masks and which is the best hygiene protocol for them. Two studies [16,26] used scanning electron microscopy (SEM) and ATP analysis with a bioluminometer, concluding that the best hygiene protocol is the use of a soluble tablet containing sodium carbonate and sodium sulfate, followed by brushing. The other two studies [15,17] showed a statistically significant difference (*p* < 0.05) in the immersion of the aligners in an ultrasonic bath and cationic detergent for the first one and chlorhexidine for the second one. It appears from all studies that the combination of a mechanical component with the aligner cleaning devices makes hygiene itself more effective.

In the second group regarding home oral hygiene, a statistically significant (*p* < 0.05) growth of microbial flora was found in those wearing fixed versus invisible orthodontics in six out of seven studies [18,20,21,22,23,25]. Various indices were analyzed. GI increased in fixed orthodontic wearers (*p* = 0.001) [22] and decreased by 86% (*p* = 0.015) in invisible orthodontic wearers [19]. PD, BOP, and PI, in two studies, also decreased in favor of invisible orthodontics [18,25]; however, two other studies [20,21] showed no statistically significant differences between indices.

**Table 2:** Outcome and results of included studies concerning aligner cleaning techniques.

Author (Year)	Protocol	Outcome	Results
Levrini et al. (2015) [26]	2 weeks: rinse for 15 s with cold running water twice a day (control group); 2 weeks: soak for 30 min in cold water with dissolved effervescent tablet containing sodium carbonate and sodium sulfate. Before reusing aligners, clean for at least 30 s with soft-bristled toothbrush and medium-abrasiveness toothpaste (RDA < 150); 2 weeks: brush for at least 30 s with soft-bristled toothbrush and medium-abrasiveness toothpaste (RDA < 150).	Microbiological analysis: assessment of the amount of plaque by scanning electron microscopy (SEM).	On exterior surfaces, Group 3 showed better cleaning results than the control group (Group 1). The best result was found in Group 2. At the level of interior surfaces, no difference was found. Bacterial contamination was found to be mostly organic, only occasionally inorganic with crystallized tartar. Only one species of spheroidal microorganisms was found.
Levrini et al. (2016) [16]	2 weeks: rinse for 15 s with cold running water each time the aligners are removed (control group); 2 weeks: brush for at least 30 s with soft-bristled toothbrush and low-abrasiveness toothpaste (RDA < 100); 2 weeks: soak for 20 min in cold water with dissolved effervescent tablet containing sodium carbonate and sodium sulfate. Before reusing the aligners, clean for at least 30 s with soft-bristled brush and low-abrasiveness toothpaste (RDA < 100).	Microbiological analysis: assessment of bacterial concentration by analyzing the amount of ATP using a bioluminometer. Bacterial concentration is expressed in RLU (relative light units).	The mean values of bacterial concentration are: Group 1 = 583 RLU Group 2 = 188 RLU Group 3 = 71 RLU Group 3, treated with tablets and surface brushing, had the lowest median value of bacterial concentration, while the control group (Group 1) had the highest. The median values for each group are: Group 1 = 518 RLU Group 2 = 145 RLU Group 3 = 64 RLU The bacterial concentration of Group 3 was found to be statistically lower than Group 1 (*p* = 0.0003).
Lombardo et al. (2017) [17]	2 weeks: rinse with water; 2 weeks: immersion in sonic bath with water; 2 weeks: immersion in ultrasonic bath with water; 2 weeks: immersion in water bath with anionic detergent; 2 weeks: immersion in sonic bath with water and anionic detergent; 2 weeks: immersion in ultrasonic bath with water and anionic detergent; 2 weeks: immersion in water bath with cationic detergent; 2 weeks: immersion in sonic bath with water and cationic detergent; 2 weeks: immersion in ultrasonic bath with water and cationic detergent. The timing set was 5 min for all methods used, each repeated 2 times daily.	Microbiological analysis: observation of the presence of biofilm by scanning electron microscopy (SEM). The measurement is carried out using Grey scale.	Method 1 and Method 9 proved to be significantly different from all others. Method 1 was the least efficient, while Method 9 was statistically the most effective (*p* < 0.05). Overall, except for Method 1, all other mask-cleaning strategies showed ability to remove biofilm from surfaces.
Shpack et al. (2014) [15]	28 days: brushing of teeth and masks using a toothpaste containing 1400 ppm fluoride (control group); 70 days: brushing of the devices and subsequent soaking of the devices in chlorhexidine mouthwash for 15 min every evening, then rinsing with water before reinserting the mask inside the oral cavity; 70 days: vibrating bath with special crystal cleaning solution for 15 min every evening, then rinse with water before reinserting the template inside the oral cavity. At the end of the protocol, the aligners were stained by the investigators with a 1% gentian violet solution for 5 min.	Microbiological analysis: assessment of biofilm adhesion measured by photodensitometer.	Protocols 2 and 3 (chlorhexidine and vibrating bath) showed a significant reduction in bacterial biofilm adhesion (*p* < 0.001) to aligner surfaces. The protocol with chlorhexidine resulted in a 16% decrease, while the protocol with vibrating bath and cleaning crystals resulted in a 50% decrease. Using Protocol 1, which involved brushing only, it was seen that the surfaces of the posterior palatine regions and incisal edge had greater plaque accumulation.

**Table 3:** Outcome and results of included studies concerning home oral hygiene protocols of the patients.

Author (Year)	Protocol	Outcome	Results
Azaripour et al. (2015) [22]	Use of each of the following devices 3 times a day: - Toothbrush - Dental floss - Pipe cleaner	Gingival index (GI); sulcus bleeding index (SBI); approximal plaque index (API) measured with plaque detector tablet.	SBI and GI values increased in both patients with fixed orthodontics and patients with aligners, when the initiation and course of treatment are compared. However, the growth experienced by fixed orthodontic wearers is statistically significant (SBI: *p* < 0.001), (GI: *p* = 0.001), such that it can be said that they have worse gingival conditions throughout treatment than patients with clear aligners.
Caccianiga et al. (2022) [23]	Fixed orthodontics: - Toothbrush with orthodontic head - Single-tufted toothbrush - Toothbrush Invisible orthodontics: - Soft-bristled toothbrush - Flossing Patients with pathogenic flora at T1 (protocol to be repeated 2 times daily): - Sonic toothbrush - Toothbrush - Water brush	Microbiological analysis: assessment of subgingival plaque quality by scanning electron microscopy (SEM). Differentiation into pathogenic or non-pathogenic flora.	Microbiological analysis three months after the start of treatment (T1) showed that 10 of the 25 patients with fixed appliances and 3 of the 25 with aligners had pathogenic flora. These 13 patients then adopted the modified home oral hygiene protocol, and at the next 3 months (T2) none again presented pathogenic flora on microbiological analysis of plaque samples. Analyzing the data collected at T1, it can be stated that there is a statistically significant correlation between type of orthodontics (fixed) and the presence of pathogenic bacterial flora (*p* < 0.05). In fact, the 10 patients with pathogenic bacteria vs. the 3 with invisible orthodontics in whom the same conditions were detected resulted in fixed orthodontics having a *p*-value of 0.024, which is significant.
Chhibber et al. (2017) [19]	Generic home oral hygiene instructions: - Toothpaste - Sonic toothbrush - Toothbrush - Dental floss	Plaque index (PI); Gingival index; Papillary bleeding index (PBI).	Comparison of the values obtained with PI, GI, and PBI among the three orthodontic modalities included in the study (aligners, fixed braces with self-ligating attachments, and fixed braces with elastomeric attachments) showed no statistically significant differences at follow-up after 18 months from the start of treatment (T2). In contrast, after only 9 months of treatment (T1), the GI and PBI measurements of patients with invisible orthodontics appeared significantly lower than those of the other two types of treatment. In fact, aligners resulted in 86% less chance of inducing gingival inflammation (*p* = 0.015) and 90% less chance of the subject having papillary bleeding (*p* = 0.012).
Levrini et al. (2013) [25]	Use of each of the following practices 3 times a day: - Toothbrush with orthodontic head: Bass technique for 2 min - Flossing	Plaque index; Pocket probing depth (PD); Bleeding on probing (BOP); Microbiological analysis: assessment of the presence of biofilms by real-time PCR analysis.	Patients with invisible orthodontics presented a decrease in pocket depth (*p* = 0.002) and a decrease in bleeding (*p* < 0.001) after 3 months of treatment (T2), compared with the values reported at T1 (1 month after the start of treatment). A significant correlation was revealed between fixed orthodontic treatment and increased PI (*p* < 0.001) and BOP (*p* < 0.001), as well as an inverse correlation between this therapy and patient compliance with oral hygiene (*p* < 0.001). A statistically significant link between type of orthodontics and increased biofilm presence was also noted (*p* < 0.005). Thus, it is claimed that invisible orthodontics induces less bacterial plaque accumulation when compared with treatment using fixed braces; consequently, the reduced risk of periodontal disease in patients wearing clear aligners is well established.
Levrini et al. (2015) [18]	Use of each of the following practices 3 times a day: - Toothbrush with orthodontic head: Bass technique for 2 min - Flossing	Plaque index; Pocket probing depth; Bleeding on probing; Microbiological analysis: assessment of the presence of biofilms by real-time PCR analysis.	Treatment with aligners established a statistically significant difference from fixed orthodontics with regard to all parameters analyzed (PI, PD, BOP) (*p* < 0.05). The amount of biofilm present was found to be significantly higher (*p* < 0.05) in patients wearing fixed appliances. Moreover, this amount added to all periodontal indices was shown to be worse at T2, again in individuals with fixed orthodontics, than at T0 and T1.
Sfondrini et al. (2021) [21]	3 times a day: - Electric toothbrush (2 min) 1 time a day: - Floss	Plaque index; Probing pocket depth; Bleeding on probing; Microbiological analysis: quantitative and qualitative assessment of the bacterial flora constituting the biofilm by real-time PCR analysis.	PI, PPD, and BOP values showed no significant changes in both the test and control groups. The presence of the bacterial species investigated by PCR analysis did not change statistically significantly in the distribution percentage. A significant increase (*p* < 0.05) was noted in the total bacterial count from T0 (14 days after professional oral hygiene) to T1 (2 months after T0), in both the test group and the control group.
Zhao et al. 2020 [20]	After each meal/snack: - Toothbrush (Bass technique) - Flossing	Plaque index; Pocket probing depth; Bleeding on probing	PI decreased statistically significantly (*p* < 0.05) at six months after the start of treatment. BOP and PPD did not change significantly. Brushing frequency during the day increased significantly (*p* < 0.05) during the course of treatment.

### 3.3. Risk of Bias 

**Figure 2:**
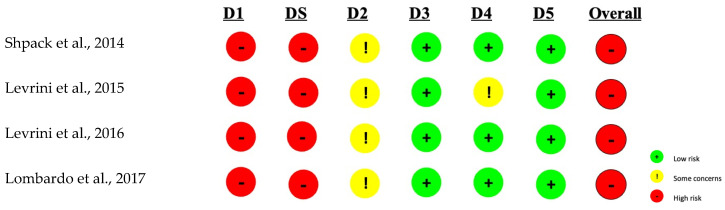
Assessment of the Risk of Bias in Modified Rob-2 Crossover Randomized Clinical Trials. The domains presented: D1: Biases arising from the randomization process; DS: Biases arising from the period and effects of travel; D2: Bias caused by deviation from the planned intervention; D3: Bias due to missing outcome data; D4: Bias in outcome measurement; D5: Bias in the selection of reported results [15,16,17,26].

Figure 2 shows the results of bias assessment in randomized crossover clinical trials included in the review. The Rob-2 rating scale (modified), an appropriate tool for this type of study, was used, according to which the overall risk of bias was found to be high. This conclusion is mainly due to the high risk found in all studies in the randomization process domain. Therefore, the level of evidence for these studies is limited.

**Figure 3:**
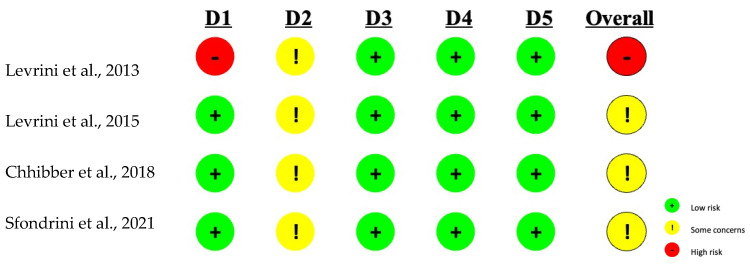
Assessment of the Risk of Bias in Rob-2 Randomized Controlled Clinical Trials. The domains presented: D1: Biases arising from the randomization process; D2: Bias caused by deviation from the planned intervention; D3: Bias due to missing outcome data; D4: Bias in outcome measurement; D5: Bias in the selection of reported results [18,19,21,25].

The Rob-2 tool was used to assess the risk of bias in RCTs. Considering the results of each individual domain for each publication, it can be concluded that the overall risk for RCTs ranges from high to moderate (Figure 3). Although three of the domains for each study had a low level of risk, the outcome was a high risk of bias in the study by Levrini et al. [25] due to the randomization process and moderate risk for studies by Levrini et al. [26], Chhibber et al. [19], and Sfondrini et al. [21]. The overall moderate risk is due to the “some concerns” rating attributed to domain 2 for each study, which was based on both participant and investigator knowledge of the intervention to which each patient was assigned. In fact, in this type of study, it was impossible to apply a blinded or double-blind trial precisely because of the nature of the intervention that was proposed to be studied.

The present assessment of the risk of bias highlights evidence with limited value, which must, however, be considered in the context of the limitations that the intervention itself presents and is not necessarily due to the methodological quality of the studies included.

The Cochrane ROBINS-I tool was used for the remaining cross-sectional observational studies included in the review. All domains had a low risk, except for biases in the measurement of outcomes, which were at moderate risk for the Azaripour et al. [22] and Zhao et al. [20] studies. This was due to the examiners’ awareness of the intervention received by the participants. The double-blind approach in these studies was impossible to apply because the measurement of outcomes involved the detection of periodontal indices, performed by experimenters who inevitably became aware of the presence of fixed or invisible orthodontics during their detection. Therefore, the overall risk according to ROBINS-I is low to moderate. In this case, as already specified for the evaluation of RCT studies, it is necessary to consider the limitations of the intervention when reading the quality of evidence.

## 4. Discussion

The use of aligners in orthodontics is becoming a widespread method to mainly treat adults, compared to the fixed techniques still used for children and adolescents, for its promising results in terms of aesthetics and oral hygiene maintenance. While the general consensus in the literature is evident in stating that fixed orthodontics promotes the accumulation of bacterial plaque due to oral hygiene difficulties [12,22,27,28,29,30], invisible orthodontics, being removable, allows an easier performance of oral hygiene maneuvers, which can be performed in the same way of a patient who does not have orthodontics.

Aligners are also proposed in patients with periodontal disease or those at risk of periodontitis: several publications claim that these patients, treated with invisible orthodontics, show no increased risk of developing gingivitis and/or periodontitis and demonstrate improved periodontal conditions [27,28,31,32], for easier removal of biofilm, which represents the main etiological cause of gingival inflammation and, if persistent, of periodontitis [33].

However, if the patient does not properly perform oral hygiene procedures for both the aligners and the oral cavity, bacterial adhesion on the surfaces of the aligners can lead to biofilm growth within two weeks, a range in which they are replaced during orthodontic treatment [15]. Another factor that causes bacterial accumulation is the presence of micro-abrasions and micro-corrugations of the plastic material of the device [3]: if not properly removed, biofilm tends to expand by exploiting the common characteristics of both dental surfaces and aligners—that is, non-exfoliation of surfaces [4].

From this review, the most effective protocols to avoid bacterial colonization and maintain oral health for aligners and oral cavities were identified. Concerning strategies for cleaning the aligners, Levrini et al. [16,26] conducted two different trials to evaluate three modes of disinfection, using two types of microbiological analysis (SEM and ATP measurement using a bioluminometer). Both publications assessed that the most effective protocol was the one using a water-soluble tablet containing sodium carbonate and sodium sulfate for immersion of the device, associated with the use of a soft-bristled toothbrush used with moderately abrasive toothpaste, to avoid altering the surface of the aligners. Considering these results, it can be concluded that the synergistic action of chemical and mechanical components is more effective than a singular component. Plus, this combination does not eliminate all bacterial residues, but it is significantly more effective than rinsing the device with water alone.

Similar outcomes emerged from the analysis performed by Lombardo et al. [17], in which nine different methods of cleaning the devices were investigated, each one applied to a pair of aligners used for two weeks. One technique, which involves rinsing under running water, has proven to be the least effective in reducing the accumulation of bacterial biofilms. The other eight cleansing modes are different combinations that can be formed between the use of a sonic bath, ultrasonic bath, cationic detergent, and anionic detergent. All of them showed the ability to reduce bacterial counts, but the results showed that the most effective protocol was the combination of an ultrasonic bath and a cationic detergent. Moreover, the association of a vibrating bath and cleansing crystals showed three times better results than immersion in a mouthwash with chlorhexidine alone [15].

Considering the abovementioned data, it can be stated that cleansing by means of chemical action of antibacterials and detergent solutions alone is not sufficient, or at least can be considerably increased by the addition of a mechanical device, e.g., a toothbrush, preferably with soft bristles, or a device that produces ultrasound [15,16,17]. Mechanical action is necessary because after two weeks of use of the aligners, the accumulation of soft plaque that initially forms has, even if only partially, turned into a semi-calcified deposit [15].

Regarding home oral hygiene, the efficacy of a home oral hygiene protocol in patients with fixed and invisible orthodontics was compared [19]. The instructions, including the use of a toothbrush, floss, and interdental brush, did not show different results in terms of periodontal preservation in the different categories of patients. Clinical indexes collected during the trial did not show any significant differences between the beginning and the end of orthodontic treatment, as well as between patients with two different types of orthodontics, thus suggesting that this protocol does not show relevant efficacy.

Another study [21] investigates home oral hygiene in invisible orthodontics without comparison with fixed devices, showing a negative result, that is, without significant improvements in oral hygiene. The protocol includes the use of an electric toothbrush three times a day and flossing once a day. Another study confirmed that the adopted protocol did not bring significant benefits to the patient [20]: in this case, the use of a manual toothbrush with the Bass technique and dental floss after each meal before reinserting the trays was evaluated. Furthermore, there was a decrease in the plaque index six months after the start of treatment, whereas the other periodontal indexes showed no difference between baseline and follow-up.

On the other hand, several publications affirmed the improvement of periodontal conditions of patients with invisible aligners after the use of specific protocols. Azaripour et al. [22] proposed the use of a manual toothbrush, floss, and pipe cleaner three times a day, daily, comparing one group of patients with fixed orthodontics and another with invisible orthodontics, and highlighting that the second group experienced a significant drop in gingival inflammation. Levrini et al. [16,26] analyzed the association of a toothbrush with an orthodontic head using the Bass technique for 2 min and dental floss, finding significantly better periodontal indexes and greater compliance in the population treated with aligners. Caccianiga et al. [23] focused on the quality of the pathogenic flora found in the oral cavity of two groups of patients with fixed and invisible orthodontics. Protocols included the use of a toothbrush with an orthodontic head, a single-tufted toothbrush, and an interdental brush for the group with fixed orthodontics and the use of a soft-bristled toothbrush and dental floss for the group with aligners. Microbiological analysis identified a greater number of patients with pathogenic flora in the fixed orthodontics group after 3 months of treatment. It is interesting to note that in this study, after the introduction of a modified protocol studied by the authors, none of the subjects in whom pathogenic bacterial flora was found no longer presented this condition—this protocol involved the use of a sonic toothbrush, water flosser, and interdental brush.

Concerning the limitations of the present review, the quality of the evidence was low despite the results obtained, mainly because of the high risk of bias due to the randomization process used in the included studies. Among the publications related to patients’ hygiene protocols, some studies were randomized controlled, while others were cross-sectional observational studies. Qualitative analysis of the evidence evidenced that randomized studies have a moderate/high risk of bias, while cross-sectional observational studies have a moderate/low risk of bias. In addition, the heterogeneity of the protocols for home oral hygiene presented in the included studies underlines the need for individually tailored effective methods to maintain daily oral hygiene care both for orthodontic devices and the oral cavity during treatment. Future directions of studies analyzing strategies for periodontal health in patients treated with aligners should thus consider specific instructions based on individual capacity, manual skill, and commitment to regular maintenance, in addition to the use of chemical agents to enhance both plaque control and device disinfection. It would be advisable to compare groups adherent to different types of protocols and habits, with the aim of exploring the effects of various combinations of procedures not only during the treatment time but also from a long-term perspective after the orthodontic therapy.

## 5. Conclusions

As studies examined in this systematic review are characterized by heterogeneity, it is not possible to determine with certainty which is the most effective protocol for adequate oral hygiene at home during treatment with invisible orthodontics. Due to methodological problems mainly related to the lack of a correct randomization process and the possibility of blinded or double-blind experiments, studies included in the present literature review are characterized by a low level of certainty, and further evidence is needed. From a clinical point of view regarding ways to keep the aligners clean during treatment, even if the singular mechanical action seems to be more effective than the chemical one, the best outcomes were obtained with the synergistic action of both mechanical and chemical agents, suggesting that it is fundamental for patients to focus on specific combinations for home aligner cleansing.

## References

[1] Shaw W.C., O’Brien K.D., Richmond S. (1991). Quality control in orthodontics: Factors influencing the receipt of orthodontic treatment. Br. Dent. J..

[2] Saccomanno S., Saran S., Laganà D., Mastrapasqua R.F., Grippaudo C. (2022). Motivation, Perception, and Behavior of the Adult Orthodontic Patient: A Survey Analysis. Biomed. Res. Int..

[3] Low B., Wilson L., Seneviratne C.J., Samaranayake L.P., Hägg U. (2011). Ultrastructure and Morphology of Biofilms on Thermoplastic Orthodontic Appliances in “fast” and “slow” Plaque formers. Eur. J. Orthod..

[4] Charavet C., Gourdain Z., Graveline L., Lupi L. (2022). Cleaning and Disinfection Protocols for Clear Orthodontic Aligners: A Systematic Review. Healthcare.

[5] Bowden G.H., Li Y.H. (1997). Nutritional influences on biofilm development. Adv. Dent. Res..

[6] Stodley P., Dodds I., Boyle J.D., Lappin-Scott H.M. (1998). Influence of hydrodynamics and nutrients on biofilm structure. J. Appl. Microbiol..

[7] Ahn S.J., Lim B.S., Lee Y.K., Nahm D.S. (2006). Quantitative determination of adhesion patterns of cariogenic streptococci to various orthodontic adhesives. Angle Orthod..

[8] Zheng J., Wang X., Zhang T., Jiang J., Wu J. (2024). Comparative characterization of supragingival plaque microbiomes in malocclusion adult female patients undergoing orthodontic treatment with removable aligners or fixed appliances: A descriptive cross-sectional study. Front. Cell. Infect. Microbiol..

[9] Nascimento M.M., Alvarez A.J., Huang X., Browngardt C., Jenkins R., Sinhoreti M.C., Ribeiro A.P.D., Dilbone D.A., Richards V.P., Garrett T.J. (2019). Metabolic profile of supragingival plaque exposed to arginine and fluoride. J. Dent. Res..

[10] Yan D., Liu Y., Che X., Mi S., Jiao Y., Guo L., Song L. (2021). Changes in the microbiome of the inner surface of clear aligners after different usage periods. Curr. Microbiol..

[11] Lucchese A., Nocini R., Lo Giudice A., Asperio P., Guglietta F., Carenzi L., Bertacci A., Donadello D., Farronato M., Maspero C. (2022). Oral and throat microbiological changes after orthodontic debonding. New Microbiol..

[12] Abbate G.M., Caria M.P., Montanari P., Mannu C., Orru G., Caprioglio A., Levrini L. (2015). Periodontal health in teenagers treated with removable aligners and fixed orthodontic appliances. J. Orofac. Orthop..

[13] Sterne J.A.C., Savović J., Page M.J., Elbers R.G., Blencowe N.S., Boutron I., Cates C.J., Cheng H.Y., Corbett M.S., Eldridge S.M. (2019). RoB 2: A revised tool for assessing risk of bias in randomised trials. BMJ.

[14] Sterne J.A., Hernán M.A., Reeves B.C., Savović J., Berkman N.D., Viswanathan M., Henry D., Altman D.G., Ansari M.T., Boutron I. (2016). ROBINS-I: A tool for assessing risk of bias in non-randomised studies of interventions. BMJ.

[15] Shpack N., Greenstein R.B., Gazit D., Sarig R., Dan Vardimon A. (2014). Efficacy of three hygienic protocols in reducing biofilm adherence to removable thermoplastic appliance. Angle Orthod..

[16] Levrini L., Mangano A., Margherini S., Tenconi C., Vigetti D., Muollo R., Abbate G.M. (2016). ATP Bioluminometers Analysis on the Surfaces of Removable Orthodontic Aligners after the Use of Different Cleaning Methods. Int. J. Dent..

[17] Lombardo L., Martini M., Cervinara F., Spedicato G.A., Oliverio T., Siciliani G. (2017). Comparative SEM analysis of nine F22 aligner cleaning strategies. Prog. Orthod..

[18] Levrini L., Novara F., Margherini S., Tenconi C., Raspanti M. (2015). Scanning electron microscopy analysis of the growth of dental plaque on the surfaces of removable orthodontic aligners after the use of different cleaning methods. Clin. Cosmet. Investig. Dent..

[19] Chhibber A., Agarwal S., Yadav S., Kuo C., Upadhyay M. (2018). Which orthodontic appliance is best for oral hygiene? A randomized clinical trial. Am. J. Orthod. Dentofac. Orthop..

[20] Zhao R., Huang R., Long H., Li Y., Gao M., Lai W. (2020). The dynamics of the oral microbiome and oral health among patients receiving clear aligner orthodontic treatment. Oral Dis..

[21] Sfondrini M.F., Butera A., Di Michele P. (2021). Microbiological Changes during Orthodontic Aligner Therapy: A Prospective Clinical Trial. Appl. Sci..

[22] Azaripour A., Weusmann J., Mahmoodi B., Peppas D., Gerhold-Ay A., Van Noorden C.J.F., Willershausen B. (2015). Braces versus Invisalign®: Gingival parameters and patients’ satisfaction during treatment: A cross-sectional study. BMC Oral Health.

[23] Caccianiga P., Nota A., Tecco S., Ceraulo S., Caccianiga G. (2022). Efficacy of Home Oral-Hygiene Protocols during Orthodontic Treatment with Multibrackets and Clear Aligners: Microbiological Analysis with Phase-Contrast Microscope. Healthcare.

[24] Page M.J., McKenzie J.E., Bossuyt P.M., Boutron I., Hoffmann T.C., Mulrow C.D., Shamseer L., Tetzlaff J.M., Akl E.A., Brennan S.E. (2021). The PRISMA 2020 statement: An updated guideline for reporting systematic reviews. BMJ.

[25] Levrini L., Abbate G.M. (2013). Assessment of the periodontal health status in patients undergoing orthodontic treatment with fixed or removable appliances. A microbiological and preliminary clinical study. Cumhur. Dent. J..

[26] Levrini L., Mangano A., Montanari P., Margherini S., Caprioglio A., Abbate G.M. (2015). Periodontal health status in patients treated with the Invisalign® system and fixed orthodontic appliances: A 3 months clinical and microbiological evaluation. Eur. J. Dent..

[27] Miethke R.-R., Vogt S. (2005). A comparison of the Periodontal Health of Patients during Treatment with the Invisalign® System and with Fixed Orthodontic Appliances. J. Orofac. Orthop..

[28] Miethke R., Vogt S. (2007). A comparison of the Periodontal Health of Patients during Treatment with the Invisalign® System and with Fixed Lingual Appliances. J. Orofac. Orthop..

[29] Cantekin K., Celikoglu M., Karadas M., Yildirim H., Erdem A. (2011). Effects of orthodontic treatment with fixed appliances on oral health status: A comprehensive study. J. Dent. Sci..

[30] Pardo A., Baccini F., De Manzoni R. (2023). Removal of bacterial biofilm in patients undergoing fixed orthodontic treatment: A literature review. J. Appl. Cosmetol..

[31] Faccioni P., Butera A., Bazzanella S. (2023). 3D Evaluation of Upper Airway Morphological Changes in Growing Patients with Class II Malocclusion Using Sander Bite Jumping Appliance. Appl. Sci..

[32] Faccioni P., Sacchetto L., Sinigaglia S. (2023). An improvement of upper airway flow in patients treated with rapid maxillary expansion: A Cone Beam Computed Tomography study. J. Appl. Cosmetol..

[33] Löe H. (1965). Physiology of the gingival pocket. Acad. Rev. Calif. Acad. Periodontol..

